# Bulk and local structures of metal–organic frameworks unravelled by high-resolution electron microscopy

**DOI:** 10.1038/s42004-020-00361-6

**Published:** 2020-08-06

**Authors:** Lingmei Liu, Daliang Zhang, Yihan Zhu, Yu Han

**Affiliations:** 1 Physical Sciences and Engineering Division, Advanced Membranes and Porous Materials Centre, King Abdullah University of Science and Technology (KAUST), Thuwal, 23955-6900 Saudi Arabia; 2 Multiscale Porous Materials Centre, Institute of Advanced Interdisciplinary Studies, Chongqing University, Chongqing, 400044 PR China; 3 School of Chemistry and Chemical Engineering, Chongqing University, Chongqing, 400044 PR China; 4 Department of Chemical Engineering, Zhejiang University of Technology, Hangzhou, 310014 PR China

**Keywords:** Solid-state chemistry, Metal-organic frameworks, Imaging techniques

## Abstract

The periodic bulk structures of metal–organic frameworks (MOFs) can be solved by diffraction-based techniques; however, their non-periodic local structures—such as crystal surfaces, grain boundaries, defects, and guest molecules—have long been elusive due to a lack of suitable characterization tools. Recent advances in (scanning) transmission electron microscopy ((S)TEM) has made it possible to probe the local structures of MOFs at atomic resolution. In this article, we discuss why high-resolution (S)TEM of MOFs is challenging and how the new low-dose techniques overcome this challenge, and we review various MOF structural features observed by (S)TEM and important insights gained from these observations. Our discussions focus on real-space imaging, excluding other TEM-related characterization techniques (e.g. electron diffraction and spectroscopy).

## Abstract (web summary)

While the bulk structures of metal–organic frameworks can be solved by diffraction-based techniques, characterization of their local structures has been lacking. Here the authors review recent advances in (scanning) transmission electron microscopy that have made it possible to probe the local structures of MOFs at atomic resolution.

## Introduction

Metal–organic frameworks (MOFs) comprise metal centres/clusters with organic coordinating ligands and are characterized by their designable topologies, porosity, and functionalities^1,2^. Studies on the structure–property relationship of MOFs are mainly based on their periodic crystallographic structures solved by diffraction-based techniques. In fact, the local non-periodic structures of MOFs—such as crystal surfaces, boundaries/interfaces, guest molecules, and point or extended defects—also have important effects on the properties of mass transport, sorption, and catalysis; for example, defects and interfaces severely affect how MOF membranes separate gases^3,4^. However, probing local structures in MOF crystals with high spatial resolution is difficult, which poses a challenge to establishing a correlation between the properties of a MOF and its local structures with atomic precision.

High-resolution transmission electron microscopy (HR-TEM) is the most widely used tool for imaging non-periodic local structures of crystalline materials, which are invisible in diffraction^5,6^. Unfortunately, MOFs are extremely sensitive to the electron beam irradiation and their structures can be easily damaged during HR-TEM imaging^7–9^. In fact, high-resolution imaging of electron beam-sensitive materials (not only MOFs) is one of the most difficult applications of TEM^10–13^. For a beam-sensitive material, the structural information contained in its HR-TEM image (or less strictly, the ‘resolution’ of the image) is not determined by the resolving power of the microscope but by the beam stability of the material—that is, how much structural integrity can be preserved under the imaging conditions. Most MOFs are immediately and completely amorphized under typically used HR-TEM conditions and, therefore, HR-TEM is conventionally considered unsuitable for MOFs.

**Fig. 1:**
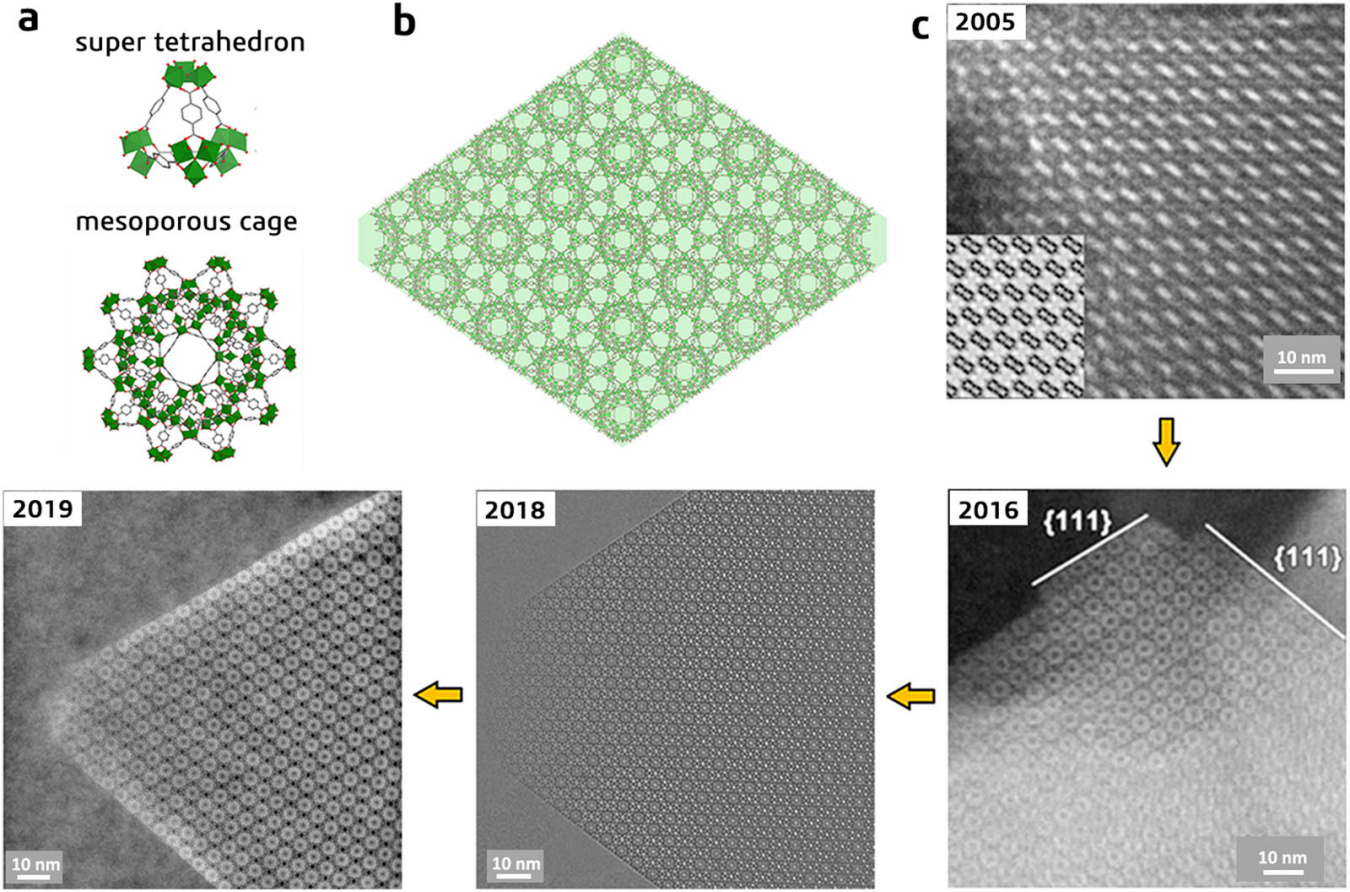
Exemplification of how the advancement in electron microscopy has led to the continuous improvement of MOF image resolution. **a** Structural models of the building units (super tetrahedron and mesoporous cage). **b** Schematic illustration of the [110] projected structure of MIL-101 with the typical truncated octahedral crystal morphology imposed. **c** A series of electron microscopy images of MIL-101 acquired in different years: HR-TEM image in 2005 (adapted with permission from ref. ^23^. © 2005 American Chemical Society); ADF-STEM image in 2016 (adapted with permission from ref. ^46^. © 2016 Wiley-VCH Verlag GmbH & Co. KGaA, Weinheim); low-dose HR-TEM image obtained with the assistances of a DDEC camera in 2018 (published in 2019) (adapted with permission from ref. ^36^. © 2019 American Chemical Society), iDPC-STEM image in 2019 (unpublished), clearly showing that the image resolution is continuously improved with the advancement of the imaging techniques.

The mechanisms of electron beam-induced structural damage are complex and vary with the material, mainly including knock-on damage, heating effects, and radiolysis^14,15^. Performing HR-TEM with a low accelerating voltage (low-voltage TEM) can reduce the energy of the incident electron beam, thereby reducing knock-on damage, which is particularly helpful for imaging low-dimensional materials such as carbon nanotube and graphene^16,17^. However, low-voltage TEM is not a good choice for MOFs, because the main structural damage mechanism of MOFs is not ‘knock-on’ but the radiolysis effect of the electron beam, which is more pronounced at lower voltages^18,19^. Low-voltage TEM also has other shortcomings, including poor resolution and weak penetration. Performing HR-TEM at cryogenic temperatures (cryo-TEM) is another option that has been proved to help improve the beam stability of the specimen; however, when the specimen is extremely sensitive, like MOFs, the limited improvement is not sufficient to resolve the structural damage issue^20,21^. Given that the beam-induced damage is a result of electron/specimen interactions, performing HR-TEM with a sufficiently low electron dose (low-dose TEM) to capture the structure before damage occurs is, in principle, a more general solution to this issue, regardless of the exact damage mechanism. This brings up the following question: How much electron dose is ‘sufficiently low’ for MOFs? In an earlier study^22^, we evaluated the stability of MOF ZIF-8 under a 300-kV electron beam by monitoring its electron diffraction patterns; we found that ZIF-8 began to lose crystallinity when the cumulative electron dose reached ∼25 e^−^ Å^−2^. We realized from subsequent research that the stability of MOFs varies with structure, composition, crystal size, and crystal orientation, but that they are generally highly sensitive and can only withstand extremely low electron doses (5–30 e^−^ Å^−2^). These values roughly define the maximum available electron dose during HR-TEM, which is approximately two orders of magnitude lower than the electron dose normally used by conventional HR-TEM. At the magnifications required for atomic-resolution images, such a low dose is equivalent to only a few electrons per pixel. Conventional charge-coupled device cameras are unable to work under such conditions because they usually require hundreds of electrons per pixel to produce acceptable signal-to-noise (SNR) ratios. This explains why in the initial attempts to image MOFs using HR-TEM, the obtained images showed limited resolution that could only resolve the primary channels/cages at best (see Fig. 1)^23^. The reason for this is that the minimum dose used in conventional HR-TEM still far exceeds the threshold, thereby resulting in structural damage to the MOF framework and, thus, loss of high-frequency information in the image.

The invention of direct-detection (DD) cameras provide an effective solution to this challenge^24–26^. DD cameras have been combined with cryo-TEM, which led to a breakthrough in structural biology with significantly improved three-dimensional voxel resolution for non-periodic protein structures^27–29^. Han’s group first used a DD camera equipped with an electron-counting function to successfully perform HR-TEM imaging of extremely sensitive crystalline materials^22^. The reason for the success is that the high detective quantum efficiency of a DD electron-counting (DDEC) camera enables HR-TEM to be performed with a sufficiently low electron beam dose to preserve the structure of sensitive materials. The group also developed a suite of methods to address a few practical challenges associated with the extremely low electron dose used^30^. The challenges include the rapid search for a crystal zone axis to minimize beam irradiation, the precise alignment of the image to fully retrieve the high-resolution information, and the accurate determination of the defocus value to process the image to be more interpretable by correcting the ‘contrast inversion’ caused by the contrast transfer function (CTF) of the objective lens. Combining the use of a DDEC camera with these methods, they successfully acquired atomic-resolution TEM images of various bulk and local structures in different MOFs^30–37^. The technical details of these methods can be found in the original publication^30^ and are not included here as they are beyond the scope of this article.

Scanning transmission electron microscopy (STEM), which uses a focused electron beam (probe) to form images, has also been used to study MOFs^38–40^. Depending on the scattering angles of the electrons used, STEM can produce bright-field (BF), annular BF, annular dark-field (ADF), and high-angle ADF (HAADF) images^41,42^. STEM has been proven to be more destructive than TEM when imaging low-Z materials at an identical electron dose due to the high instantaneous dose rate of STEM, thereby suggesting that it is more challenging to image MOFs with STEM than with TEM^43,44^. This is particularly true for HAADF-STEM, which utilizes only a rather small fraction of the incident electrons (those scattered to high angles), thereby requiring a higher electron dose to produce sufficient signals in the image compared with other imaging modes^45^. On the other hand, HAADF-STEM is sensitive to the variation of atomic number (Z) in the specimen and its image contrast is approximately proportional to Z^2^; the higher image contrast makes HAADF more tolerant to noise than other imaging methods, and particularly useful for identifying heavy elements in a matrix of material composed of light elements. Early work has shown that although the image resolution is limited due to beam damage, HAADF-STEM can be used to identify the distribution of metal nodes and heavy dopants in MOFs (Fig. 1)^46–50^.

Recently, an emerging STEM technique—integrated differential phase-contrast STEM (iDPC-STEM)—has been attracting widespread attention^51–53^. In comparison with standard BF and dark-field STEM modes under the same probe conditions, iDPC-STEM exhibits the best SNR ratio, due to its highest utilization of incident electrons and the integration process of the vector field obtained by DPC (see ref. ^54^ for theoretical details). Moreover, iDPC-STEM enables the simultaneous imaging of heavy and light elements and its image contrast can be approximately interpreted as the electrostatic potential field of the thin specimen. These advantages make iDPC-STEM an ideal low-dose technique for avoiding beam damage. Preliminary experimental results have demonstrated its potential in imaging sensitive materials, including MOFs (Fig. 1).

Figure 1 presents a series of electron microscopy images of MIL-101 acquired in different years^23,36,46,55^, clearly illustrating that with the advancement of imaging techniques, the continuously improved image resolution leads to the revelation of increasingly more structural details: it was difficult to identify the mesoporous cage in the initial study, but currently it is already easy to resolve the smaller super-tetrahedral building units. In this article, we review the progress in high-resolution (scanning) transmission electron microscopy ((S)TEM) imaging of MOF materials in recent years, discuss new understanding and insights on MOF structures (particularly their local structures) brought about by these technological advances, and also provide our perspectives and outlook to this subject.

## High-resolution electron imaging of MOFs

Due to the electron beam sensitivity of MOFs, the number of high-resolution (S)TEM studies on MOFs is limited. Most studies focus on a few relatively stable or mesoporous MOFs, such as ZIF-8, UiO-66, MOF-74, MIL-101, and NU-1000. As summarized in Table 1, all these imaging experiments were conducted at high accelerating voltages (200 or 300 kV), except for a few earlier studies; moreover, atomic resolution (<3 Å) had not been achieved until recently, when ultralow-dose techniques based on DDEC cameras or iDPC-STEM were employed. In the following sections, we classify these studies into different categories for detailed discussions.

**Table 1:** Successful examples of high-resolution electron microscopy imaging of MOFs.

Materials	Methods	Imaging conditions	Information transferred	Structures studied	Reference/Year
MIL-101					
MIL-101	HR-TEM	200 kV	Mesopore	Bulk	^23^/2005
	HR-TEM	300 kV, 8 e^−^ Å^−2^	2.5 Å	Bulk and surface	^36^/2019
	iDPC-STEM	300 kV, 54 e^−^ Å^−2^	4.7 Å	Bulk	^93^/2020
	iDPC-STEM	200 kV, 84 e^−^ Å^−2^	2 Å	Bulk	Unpublished (Fig. 7a)
Pt@MIL-101	ADF-STEM	200 and 300 kV, 10 pA	1.6 nm	Guest species	^46^/2016
CsPbI_3_@MIL-101	HAADF-STEM	300 kV, 2 pA	Mesopore	Guest species	^90^/2019
Ag-MIL-100	HAADF-STEM	300 kV, 162 e^−^ Å^−2^	8.17 Å	Bulk, interface, and guest species	^76^/2017
MOF-74/IRMOF-74	HR-TEM	120 kV	Mesopore	Bulk	^56^/2012
	ADF-STEM	300 kV	Micropore	Bulk	^38^/2014
	ADF-STEM	300 kV	2.9 Å	Bulk	^59^/2015
Ni-CAT-1	HR-TEM and ADF-STEM	120 kV and 60 kV	Mesopore	Bulk	^57^/2012
MOF-5	(cryo) HR-TEM	80 kV	Micropore	Bulk	^20^/2012
PCN-333	HR-TEM	200 kV	10 Å	Bulk	^58^/2015
UiO-66	HR-TEM	200 kV	6 Å	Bulk	^9^/2013
	HR-TEM	300 kV, 12 e^−^ Å^−2^	1.6 Å	Bulk and surface	^30^/2018
	HR-TEM	300 kV, 12 e^−^ Å^−2^	2 Å	Defects	^35^/2019
	iDPC-STEM	200 kV, 340 e^−^ Å^−2^	1.4 Å	Bulk	Unpublished (Fig. 7b)
ZIF-8					
ZIF-8	HR-TEM	300 kV, 4.1 e^−^ Å^−2^	2.1 Å	Bulk, surface, and interface	^22^/2017
CO_2_-ZIF-8	(cryo) HR-TEM	300 kV, 7 e^−^ Å^−2^	1.86 Å	Guest species	^91^/2019
Protein-ZIF-8	(cryo) HR-TEM	300 kV, 5 e^−^ Å^−2^	Crystal lattice	Bulk	^21^/2020
MXene-derived PMOF	HR-TEM	300 kV	<3 Å	Bulk	^60^/2019
NU-1000					
NU-1000	ADF-STEM	300 kV	Mesopore	Bulk	^92^/2017
Mn_12_Ac@NU-1000	HR-TEM	300 kV, 8 e^−^ Å^−2^	3 Å	Guest species	^34^/2019

### Bulk structures

Almost all MOFs studied by HR-(S)TEM have known crystal structures that are solved by X-ray diffraction. In this sense, imaging the bulk structures of MOFs does not provide additional structural information. However, imaging known bulk structures is necessary because they are the references for determining ‘safe’ imaging conditions, thereby ensuring that the observed unknown local structures are inherent and not caused by the beam damage effect. Note that it is meaningless to image local structures under conditions that can damage the bulk structure.

**Fig. 2:**
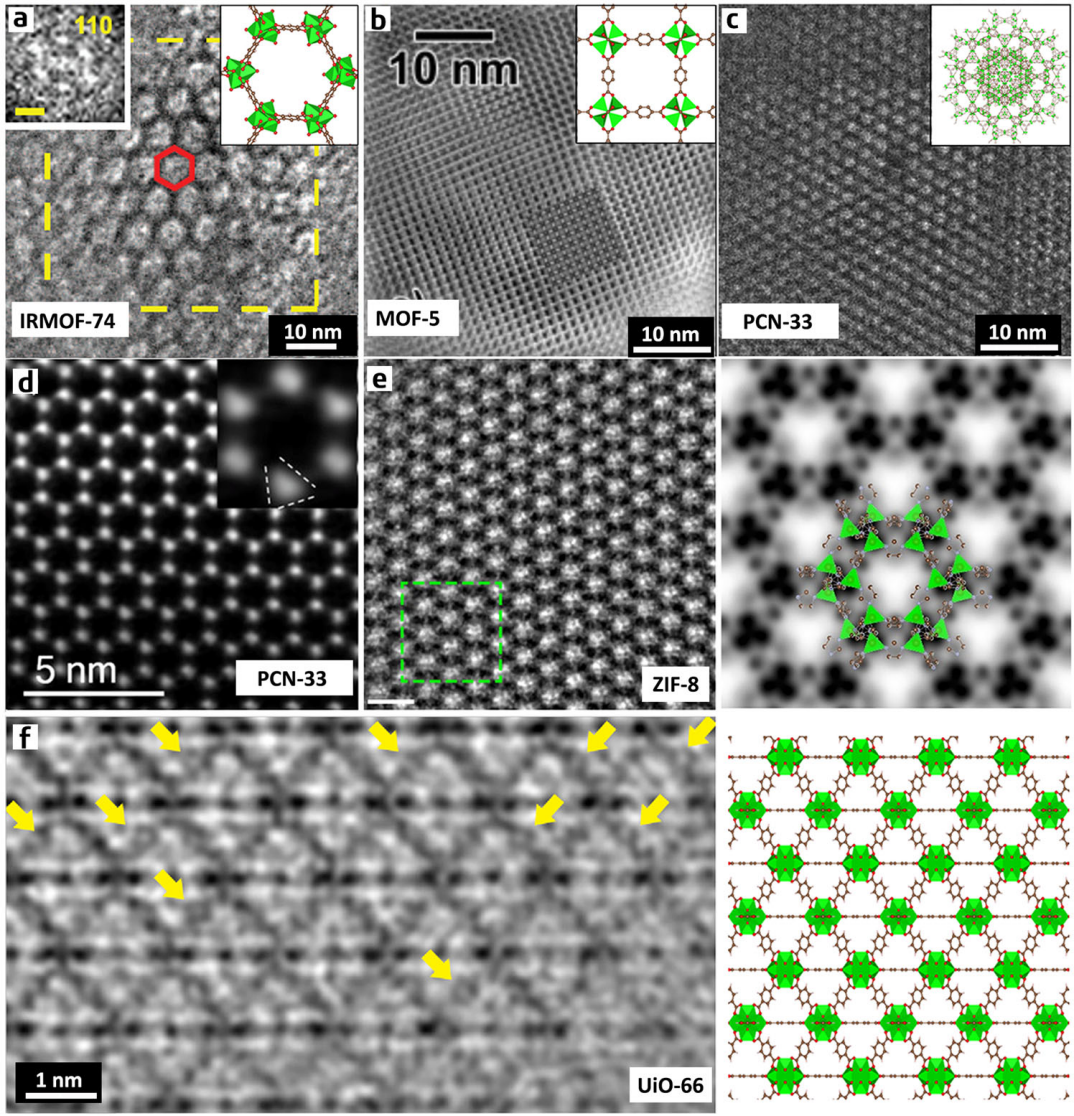
Bulk structures of various MOFs. HR-TEM images: **a** Isoreticular MOF-74 (IRMOF-74) (adapted with permission from ref. ^56^. © 2012 AAAS), **b** MOF-5 (adapted with permission from ref. ^20^. © 2017 Elsevier), and **c** PCN-333 (adapted with permission from ref. ^58^. © 2015 Springer Nature). The corresponding structural model is inset in each panel. The limited image resolution cannot reveal structural details other than the primary channels. **d** ADF-STEM image of MOF-74 processed by Fourier filtering, in which the triangular shape of the Zn clusters can be vaguely recognized, but individual Zn atomic columns cannot be resolved (adapted with permission from ref. ^59^. © 2015 Wiley-VCH Verlag GmbH & Co. KGaA, Weinheim). **e** Low-dose HR-TEM image of ZIF-8 along the [111] zone axis: (left) the denoised raw image, in which the scale bars represent 2 nm; (right) symmetry-imposed, lattice-averaged image overlaid with a structural model of ZIF-8. In the processed image, Zn columns and two types of imidazole rings can be identified (adapted with permission from ref. ^22^. © 2017 Springer Nature). **f** Low-dose HR-TEM image of UiO-66 along the [011] zone axis processed by CTF-correction and the corresponding structural model. The high resolution of the image allows the benzene rings in the BDC linkers to be resolved, as indicated by the yellow arrows (adapted with permission from ref. ^30^. © 2018 AAAS).

It is due to beam damage that electron microscopy imaging of MOFs in earlier studies could only achieve nano-scale resolution even in the best cases. The images usually only showed the ordered arrangement of the main channels or cavities but could not reveal more structural details in the frameworks. Deng et al. used HR-TEM to image a series of isoreticular MOF-74 (termed IRMOF-74) at 120 kV; their study observed hexagonally arranged channels (Fig. 2a)^56^. The *d*-spacing measured from the image (5.57 nm) was in good agreement with that derived from the X-ray crystal structure analysis (5.69 nm), thereby providing additional evidence of pore enlargement by using a new ligand. Hmadeh et al. imaged a two-dimensional MOF, Ni-CAT-1, at 120 kV to verify its ordered porous structure at the nanometre scale^57^. Wiktor et al. combined low voltage (at 80 kV) with cryo-TEM (at the liquid nitrogen temperature) in order to minimize knock-on and heating effects simultaneously^20^. They successfully acquired an image along the <100> zone axis of MOF-5, which matched reasonably well with the simulated HR-TEM image, showing a cubic lattice consisting of Zn clusters after image filtering (Fig. 2b). In these earlier studies, low-voltage conditions (80–120 kV) were used to reduce the ‘knock-on’ damage, but the limited image resolution suggested that ‘knock-on’ might not be the major damage mechanism of MOFs. Performing HR-TEM with high accelerating voltages (200–300 kV) can alleviate the radiolysis effect that was subsequently recognized as the dominant factor in the beam damage of MOFs^15,19^. However, without the ability to produce images using a ‘sufficiently low’ electron dose, it is also impossible to achieve atomic resolution for MOFs under high-voltage conditions. For example, Feng et al. performed HR-TEM for PCN-333 at 200 kV and the obtained image exhibits a limited resolution of ~1 nm^58^. This resolution is sufficient to identify the mesopores of 3.7 nm and helps determine the space group of PCN-333 to be *Fd*-3*m* (Fig. 2c). The processed, symmetry-imposed image matches well with the projected electrostatic potential of PCN-333 calculated from the proposed structural model, thereby supporting the accuracy of the model.

STEM has also been used to image MOFs. Mayoral et al. reported the ADF-STEM image of Zn-MOF-74 in 2014 and improved the resolution to 2.9 Å 1 year later^38,59^. In the Fourier-filtered image, the triangular shape of the Zn clusters (triplets) can be vaguely recognized, but individual Zn atomic columns that are 2.1 Å apart from each other in the [001] projection cannot be separated (Fig. 2d). Further, organic linkers could not be identified in these dark-field images. ADF-STEM and HAADF-STEM were also used to study various MIL-101 samples (discussed in the following sections), but the obtained image resolution is lower than that obtained for MOF-74; this is likely because MIL-101 is rather prone to structural contraction under the electron beam, thereby losing atomic-scale ordering.

In all these studies, irrespective of what imaging modes and conditions were used, atomic-resolution images could not be obtained for MOFs. That fact that the ‘resolution’ of the image (defined by the smallest *d*-value of the peaks in its Fourier transform) is always much lower than the actual resolving power of the microscope used indicates that structural damage is the limiting factor for the image ‘resolution’. On the other hand, the image ‘resolution’ can be used as an indicator of how well the MOF structure is preserved during imaging.

A significant breakthrough came in 2017, when Han’s group reported the use of a DDEC camera to facilitate the HR-TEM of a MOF for the first time^22^. In this work, they used a regular non-Cs-corrected electron microscope to image MOF ZIF-8 at 300 kV and room temperature. The high QDE of the DDEC camera enabled HR-TEM imaging to be performed with an electron dose as low as ~4 e^−^ Å^−2^, thereby ensuring minimal structural damage during imaging. The obtained image involves structural information transferred up to 2.1 Å, indicating a well-preserved crystal structure. Such a high resolution enables individual atomic columns of Zn and two types of imidazole rings (edge-on and face-on) to be resolved along the [111] direction in the processed image (Fig. 2e). These results demonstrate the great potential of DDEC cameras in the HR-TEM of extremely sensitive materials. It is worth noting that for crystalline materials, HR-TEM images need to be acquired along specific directions, that is, along the zone axes of the lattice, in order to avoid overlapping of atoms in the projection image. This requirement imposes additional challenges when dealing with extremely sensitive materials because the process of finding the zone axis (zone axis alignment) that also consumes electron dose must be accomplished very quickly in order to minimize beam irradiation. The common practice in HR-TEM is to manually align the zone axis, which can easily consume an electron dose of up to hundreds of electrons per Å^2^, thereby destroying the crystal structure at this step (before image acquisition). In the initial report, this challenge was circumvented by sampling a large number of randomly oriented crystals and selecting only a few crystals that happened to be oriented along the zone axis for further analysis. However, this is not an ideal solution to this challenge because it is rather inefficient.

Han’s group solved the problem of how to quickly align the zone axis 1 year later^30^. They developed a simple program to achieve an automatic one-step alignment of the zone axis, which can limit the total dose required for the zone axis alignment process to be <1 e^−^ Å^−2^, thereby effectively avoiding beam damage during this step and significantly increasing the probability of obtaining useful HR-TEM images. They also developed an image filtering method that enables the precise alignment of a series of short-exposure frames in an image stack in order to avoid the loss of high-resolution information caused by specimen drift during HR-TEM. Moreover, they proposed a new method to determine the defocus value of the obtained low-dose HR-TEM image, so that the image can be processed through CTF-correction to be more interpretable. Thus, based on the use of DDEC cameras, this set of methods further advanced the atomic-resolution TEM imaging of MOFs and other beam-sensitive materials to an almost-routine process. In this study, MOF UiO-66 was used as an example to show the effectiveness of this set of methods. The processed image of the [011] zone axis perfectly matches the structural projection with information transfer of 1.5 Å. In the image, the triangular channels encompassed by three Zr_6_O_8_ clusters and three 1,4-benzenedicarboxylic acid (BDC) linkers are identified, atomic columns of Zr within the clusters are distinguished, and most impressively, benzene rings with face-on configurations in the BDC linkers are resolved (Fig. 2f). The HR-TEM images also revealed that the octahedral Zr_6_O_4_(OH)_4_ clusters in UiO-66 become distorted upon heating to 300 °C, which is in agreement with previous studies that speculated that a thermal treatment leads to the conversion of Zr_6_O_4_(OH)_4_ clusters to Zr_6_O_6_ clusters through dihydroxylation. Although the Zr–Zr distances measured from the images are not as accurate as those determined by EXAFS, due to pixel-size limitations, HR-TEM provides the most direct evidence for an atomic-scale structure deformation in a MOF.

Recently, Wu et al. synthesized porphyrin-based MOF nanosheets using MXene V_2_CT_x_ (T = F, O, and OH) as a metal precursor; they successfully acquired HR-TEM images for these nanosheets with a DDEC camera. The obtained near-atomic-resolution images revealed highly ordered cages, which were in good agreement with the proposed crystal structure and simulated images^60^.

### Surface structures

Identifying the surface structure of a MOF is critical to determine its affinity, reactivity, and accessibility of internal pores^61,62^. Hmadeh et al. reported the first example of using HR-TEM to observe the surface of a MOF (activated Ni-CAT-1), but the obtained image did not match well with the simulated image and the resolution was not sufficiently high to provide much structural information^57^. Before the ultralow dose HR-TEM was available, scanning probe microscopy (SPM), such as atomic force microscopy (AFM) or scanning tunnelling microscopy, was the only high-resolution tool to characterize the surface structures of MOFs^63,64^. However, there are few reports of using SPM to study MOFs, because SPM is more suitable for studying flat and clean surfaces rather than discrete crystals^65^.

In their work that reported the use of a DDEC camera to enable HR-TEM of sensitive materials, Zhu et al. investigated the exposed (110) surfaces of rhombic dodecahedral ZIF-8 crystals^22^. They proposed two possible termination modes for the (110) surface of ZIF-8—‘zigzag’ and ‘armchair’—assuming that the surface Zn^2+^ ions are capped by 2-methylimidazole (Hmim). As the (110) surfaces are in an edge-on configuration when the ZIF-8 crystal is viewed along the [111] zone axis, their termination modes can be observed in this direction. However, because the image was acquired on an uncorrected TEM microscope at a large defocus value (~550 nm), it exhibited contrast delocalization and intense Fresnel fringes. These problems make it difficult to unambiguously determine the surface termination mode directly from the image. By comparing the Fresnel fringes of the experimental image and the simulated HR-TEM images of the two structural models, the surface termination mode was determined to be ‘armchair’, which is contrary to the conclusion made by Moh et al. from the AFM measurement^66^. Slater et al. explained this discrepancy as a consequence of different stages of crystal growth and/or a solvent effect on the equilibrium surface structure in the two studies; meanwhile, their calculations suggested that a ‘flat armchair’ configuration is the most favourable termination mode, which is slightly different from the ‘armchair’ model proposed by Zhu et al.^67,68^. Thus, the precise structure of the (110) surfaces of ZIF-8 remains controversial.

**Fig. 3:**
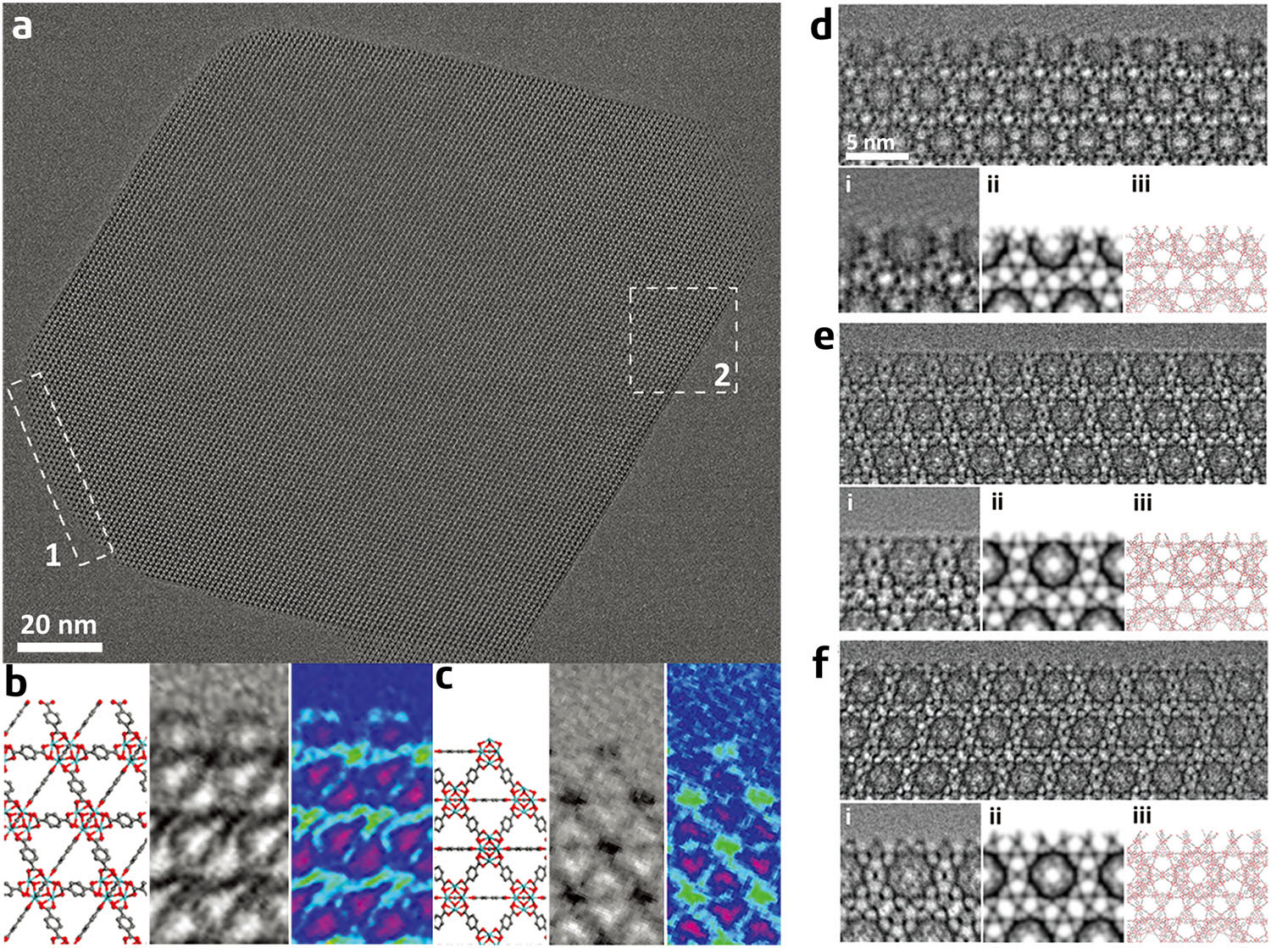
Surface structures of UiO-66 and MIL-101. **a** Low-dose HR-TEM image of a truncated octahedral UiO-66 crystal along the <011> zone axis. **b** Ligand-terminated {111} surface: (left) structural model; (middle) processed HR-TEM image by real-space averaging of area 1 in **a**; (right) the averaged image displayed in the rainbow colours to increase the visibility of the ligand contrast. **c** Metal cluster-terminated {100}/{111} kink: (left) structural model; (middle) processed HR-TEM image by real-space averaging of area 2 in **a**; (right) the averaged image displayed in the rainbow colours (adapted with permission from ref. ^30^. © 2018 AAAS). Low-dose HR-TEM images of three vacuum-heated (at 150 °C) MIL-101(Cr) samples: **d** MIL-101-HF, **e** MIL-101-NA, and **f** MIL-101-Ac, showing different ‘completeness’ of the mesoporous cages at the outermost surface. In each panel, the processed image by real-space averaging (i), the simulated projected potential map (ii), and the projected structural model (iii) are presented for comparison (adapted with permission from ref. ^36^. © 2019 American Chemical Society).

In order to obtain images that are clearer and easier to-interpret, Han’s group used Cs-corrected TEM microscope and attempted to acquire images at appropriate defocuses in subsequent studies^30–37^. They observed different surface-dependent termination modes that co-exist in UiO-66: the main exposed {111} surface is terminated with BDC linkers, whereas the truncated surface, which comprises a number of small {100} and {111} facets, is terminated with Zr clusters at the {100}/{111} kinks (Fig. 3a–c)^30^. It is worth noting that unlike previous works on the surface termination of MOFs that could only detect surface steps^63,64^ or at most only recognize metal clusters^22^, this study was the first to distinguish organic linkers from metal clusters on the MOF surface.

In a separate study, the established HR-TEM method was used to investigate the surface structures of a mesoporous MOF—MIL-101^36^. The HR-TEM observations revealed that the surface structure of MIL-101(Cr) can be tuned by using different additives (modulators) during the synthesis and by a post-synthesis thermal treatment. Specifically, three MIL-101(Cr) samples are synthesized using either HF (denoted as MIL-101-HF) or acetic acid (MIL-101-Ac) as a ‘modulator’, or no additive at all (MIL-101-NA). Among the investigated samples, three typical surface structures are identified, each one exhibiting a different degree of ‘completeness’ of the surface mesoporous cages (see Fig. 3d–f). A thermal treatment performed under vacuum, at specific temperatures, can selectively open up the surface cages without changing the bulk structure, and the required temperature is dependent on the type of additive used during the synthesis. These HR-TEM observations, in combination with in situ XRD measurements, demonstrate that the additives have a significant influence not only on the size of the crystal but also on the surface structure and the stability of the framework of MIL-101(Cr). Interestingly, solid–solid phase transformation from MIL-101 to MIL-53 caused by vacuum heating only occurs in MIL-101-HF but not in the other two samples. Although different additives are often used in the synthesis of MOFs to tune the crystal size, surface area, and product yield, this is the first time they have been found to have significant impacts on the surface structure and stability of a MOF.

### Interfacial structures

When used in various applications, MOF often forms interfaces between crystals or with other materials. Metal/MOF and oxide/MOF composites are designed to combine the sensitivity of the metal (oxide) and the selectivity of the MOF in gas sensors^69,70^; MOF crystals are often used as fillers in polymeric membranes for improved gas separation^71,72^; the attachment, assembly, and agglomeration of MOF crystals during the synthesis produces grain boundaries or MOF/MOF interfaces that influence mass transfer and diffusion properties^73,74^.

In principle, HR-(S)TEM can be utilized to investigate these interfacial structures, but there is little research on this subject. One possible reason is that many interfaces involving MOF are not compatible with the imaging or specimen preparation conditions. For example, performing atomic-resolution imaging of a noble metal/MOF interface is difficult because the electron dose required to retain the MOF structure is too low to produce a lattice image for the metal, while increasing the dose to image the metal causes damage to the MOF structure. Similarly, observing the inherent interface between the MOF filler and polymer matrix in membranes is difficult, because the TEM specimen preparation process can dislocate or damage the filler; in addition, the amorphous nature of the polymer matrix is not conducive to probing the interfacial structure with atomic precision. Currently, only MOF/MOF interfaces have been studied with HR-(S)TEM.

**Fig. 4:**
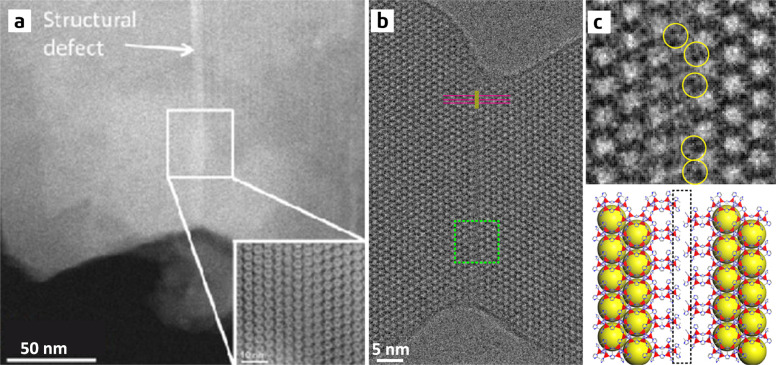
Interfacial structures observed in MIL-100 and ZIF-8. **a** HAADF-STEM image of MIL-100(Fe) along the [110] direction, with a twin plane marked by a white arrow. Inset is the enlarged review of the twin boundary (adapted with permission from ref. ^76^. © 2017 Wiley-VCH Verlag GmbH & Co. KGaA, Weinheim). **b** Low-dose HR-TEM image of the interface of two interconnected ZIF-8 crystals along the [111] zone axis. Purple lines are used to show the coherence between the lattice fringes in the two crystals that are separated by an interface represented by the yellow line. **c** CTF-corrected image of the green square area in **b**. Yellow circles highlight Zn triplets with distortions. **d** Structure model of the interface between two ZIF-8 crystals based on the HR-TEM image (adapted with permission from ref. ^22^. © 2017 Springer Nature).

MIL-100 and MIL-101 are isomorphic in structure (MTN zeotype, space group *Fd-3m*) but have different unit cell parameters due to different organic ligands. The mesoporous cages of MIL-100 are 4–5 Å smaller than the cages of MIL-101^55,75^. Mayoral et al. observed the interface between two intergrowth MIL-100 crystals using HAADF-STEM and found the image resolution to be sufficient to show the stacking of mesoporous cages on both sides of the twin plane (Fig. 4a)^76^. Meledina et al. observed a similar twinning structure in MIL-101 using HAADF-STEM^46^. The existence of such classic twinning structures in MOFs suggests that despite their complex building blocks, open frameworks, and large unit cells, MOFs have similar crystallographic behaviours as simple conventional crystals.

Rhombic dodecahedral ZIF-8 crystals have been found to self-assemble through oriented attachment between (110) interfaces, presumably driven by van der Waals attractions^77^. Such an interface was captured in the first report on the atomic resolution TEM of MOF^22^. The image reveals that two ZIF-8 crystals are interconnected through a coherent interface with an arm-to-notch configuration (Fig. 4c), thereby suggesting that the assembly of ZIF-8 crystals is unlikely driven solely by non-directional van der Waals forces. With respect to the perfect crystal structure of ZIF-8, the interfacial structure contains an additional layer of ligands, which leads to the generation of larger pores at the interface. Such an interface is presumably created by the direct adhesion of two ligand-terminated (110) surfaces via hydrogen bonding without chemical reactions. On the basis of the interfacial structure unravelled by HR-TEM, molecular dynamics simulations were performed in order to elucidate how the assembly of ZIF-8 might influence the diffusivity of various gases—including H_2_, N_2_, CO_2_, and CH_4_—in the crystals.

### Defective structures

Like other crystalline materials, MOFs contain structural defects. Defect engineering of MOFs has attracted considerable research attention, as it offers a means to create open-metal sites, modulate surface properties, and locally tune porosity, thereby having important implications for various applications of MOFs^78,79^.

There are a few studies on the direct observation of structural defects in MOFs. AFM was used to reveal the existence of screw dislocation in HKUST-1^63^, and electron microscopy was used to observe locally reorganized channels in an expanded version of MOF-74 and the twinning structure in MIL-100 and MIL-101^46,76,80^. In addition to these classic defect types, MOFs have two unique defect types, namely, ‘missing-linker’ and ‘missing-cluster’ defects, which, respectively, refer to the loss of a number of organic linkers and metal clusters, with respect to the perfect crystallographic structure^81,82^.

The existence of ‘missing-linker’ and ‘missing-cluster’ defects was speculated from abnormal macroscopic characteristics (e.g. non-stoichiometric composition and abnormal gas adsorption behaviour) and confirmed by diffraction-based methods. Both neutron powder diffraction^83^ and synchrotron single-crystal X-ray diffraction^84,85^ indicated that missing-linker defects widely exist in various UiO-66 samples. However, these studies can only determine the average degree of the loss of organic ligands, but not the spatial distribution of the resulting defects in the MOF crystal. Through the combination of electron diffraction, X-ray diffraction, and anomalous X-ray scattering, Cliffe et al. discovered that UiO-66(Hf) has nano-sized domains formed by missing-cluster defects that have a structure that follows the **reo** topology^86^. This study revealed that the defects in MOF can form short-range ordered domains; however, it failed to obtain HR-TEM images of the defective MOF, due to the beam damage issue. Fluorescence lifetime imaging was used to probe the spatial distribution of defects within individual UiO-67 crystals, but the sub-micrometre resolution was not sufficient to reveal the defect types and their exact structures^87^.

**Fig. 5:**
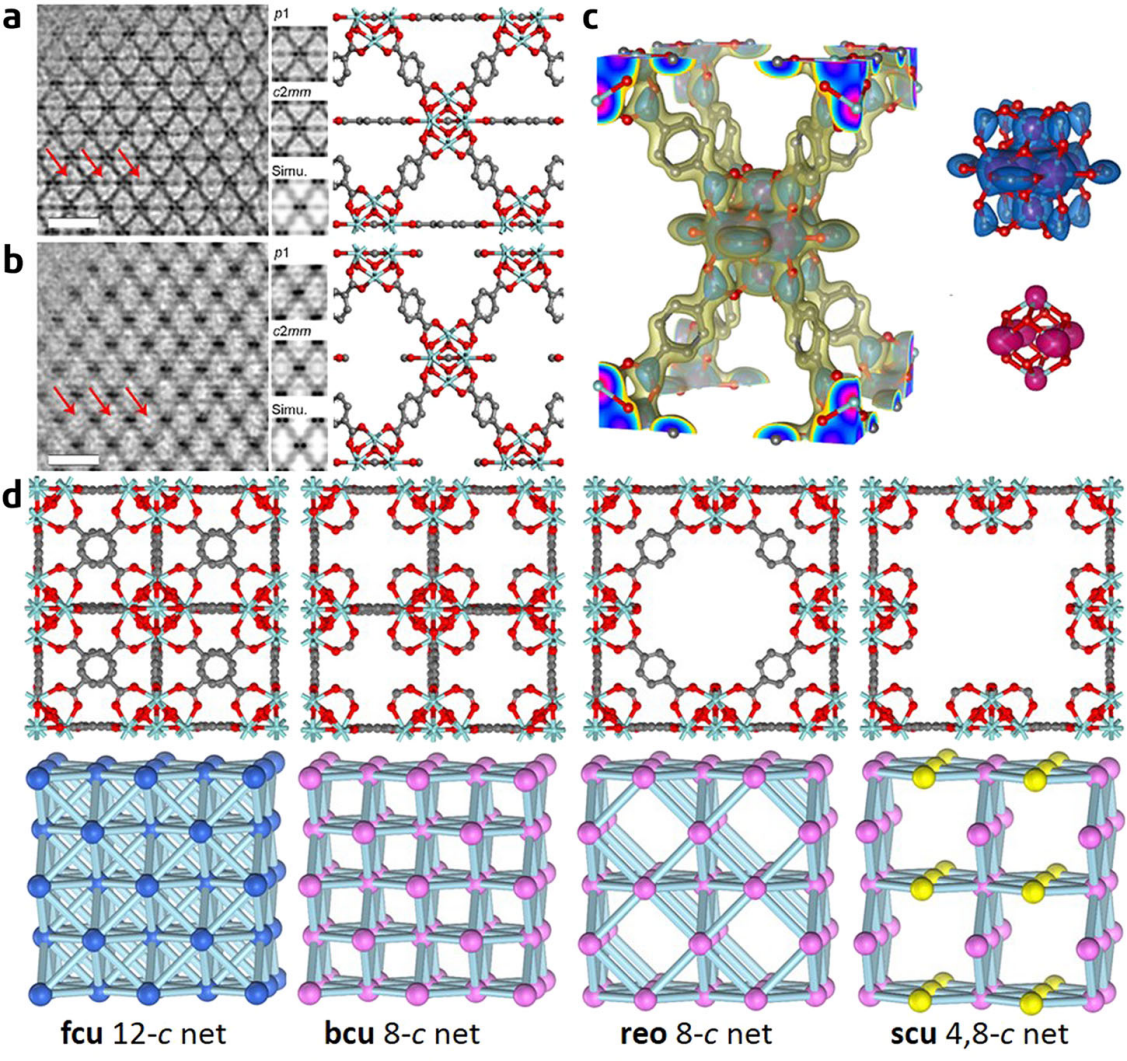
Defective structures in UiO-66. CTF-corrected low-dose HR-TEM images and structural models of **a** perfect UiO-66 (*Fm*-3*m*) and **b** the ‘missing-linker’ defect (*I*4/*mmm*). The images are along the equivalent (the [110] in **a** and the [100] in **b**) directions. Scale bars represent 2 nm. In each panel, left, CTF-corrected image; middle, from the top-down are p1-averaged image, symmetry-imposed image, and simulated projected potential; right, the projected structural model. The contrasts of the horizontally arranged BDC ligands observed in **a** are not present in **b**, as indicated by red arrows. **c** Electrostatic potential map of the ‘missing-linker’ defect reconstructed from HR-TEM images, showing an 8-connected node. Isosurfaces rendered at different thresholds are superimposed with the geometry-optimized structure model of the missing-linker defect for comparison. **d** Crystallographic structural models and corresponding topological representatives (2 × 2 × 2) of various structures unravelled by HR-TEM: 12-connected ideal UiO-66 (**fcu** net), 8-connected missing-linker defects (**bcu** net), 8-connected missing-cluster defects (**reo** net) and the 4,8-connected missing-cluster defects (**scu** net) (adapted with permission from ref. ^35^. © 2019 Springer Nature).

The DDEC-based low-dose HR-TEM enables the direct observation of structural defects in MOFs with atomic resolution. Recently, Liu et al. used this technique to investigate UiO-66 nanocrystals that were synthesized using formic acid as a modulator^35^. They found that the UiO-66 crystals contained a large number of domains, which exhibited obviously different image contrasts with apparent features of ‘missing linkers’ along the [001] and [110] directions compared with the structure of perfect UiO-66 (**fcu**, *Fm-3m*). The HR-TEM images of the perfect and defective UiO-66 along the [110] direction are shown in Fig. 5a, b, respectively; clearly, the contrasts of the horizontally arranged BDC ligands observed in Fig. 5a are not present in Fig. 5b. The authors obtained high-quality HR-TEM images from three distinct projections of such defective domains. On the basis of the three images, they successfully determined the space group (*I*4/*mmm*) and reconstructed the 3-D electrostatic potential map of the missing-linker defect using electron crystallography. The reconstructed potential map depicts an 8-connected framework, which follows the topology of a bcu net with each Zr_6_O_8_ cluster connected with eight neighbouring clusters and is apparently different from the 12-connected framework of perfect UiO-66 (Fig. 5c). The high precision of the reconstruction enables the identification of not only the Zr_6_O_8_ clusters and BDC ligands but also the terminal formates groups that substitute the missing BDC linkers to cap the open-metal sites (Fig. 5c). A series of chemical characterizations—including liquid chromatography, ^13^C NMR spectroscopy, and mass spectrometry—consistently show that a substantial amount of formates (~4 wt%) is present in the UiO-66 sample under study, thereby supporting the HR-TEM result. This is inconsistent with a previous single-crystal X-ray diffraction study that concluded that the missing-linker defects in UiO-66 are terminated by water molecules and charge-balanced by OH^−^ groups^84^. This discrepancy suggests that the type of defect capping group may vary with the synthetic conditions.

Interestingly, the HR-TEM study further revealed that in this UiO-66 sample, in addition to the bcu missing-linker defect, two types of missing-cluster defects co-exist, which follow the topologies of **reo** and **scu** nets, respectively. Similar to the **bcu** structure, the reo structure has an 8-connected framework, and it can be obtained by removing one out of four symmetry-equivalent Zr_6_O_8_ clusters along with the associated BDC linkers from each unit cell of the perfect UiO-66 structure. The scu structure, however, is more defective, with one-third of the Zr_6_O_8_ clusters 8-connected and the other two-thirds 4-connected; the scu structure can be formed by removing the Zr_6_O_8_ clusters from the bcu structure in a similar manner as done in the reo-to-fcu transition. Figure 5d presents all defective structures identified by HR-TEM and the perfect UiO-66 structure for comparison. The HR-TEM results also indicate that missing-linker defects prevail in the sample studied with domain sizes up to ~100 nm, whereas missing-cluster defects only appear in small areas that cover a few unit cells.

Unlike previous studies that could investigate only one type of defect at a time, HR-TEM imaging provided the first evidence for the coexistence of the ‘missing-linker’ and ‘missing-cluster’ defects in UiO-66. On the basis of this knowledge, it is possible to further study the evolution and transformation of different defects during the crystallization process in order to achieve precise control of defect types and their concentrations. Specifically, Liu et al. found that in their system, prolonging the crystallization time led to the ripening of UiO-66 crystals; during the ripening process, the missing-cluster defects gradually disappeared and the missing-linker defects remained. More interestingly, increasing the concentration of formic acid regulator could reverse the trend of defect evolution, that is, as the crystallization time increased, the missing-cluster defects rapidly developed. Thus, the ability to regulate the concentration of ‘missing-linker’ and ‘missing-cluster’ defects makes it possible to determine their relative catalytic activities. The results from a model reaction, the isomerization of glucose to fructose, indicate that when defect concentrations are comparable, missing-cluster defects are more catalytically active than missing-linker defects^35^.

### Guest species in MOFs

The porous, crystalline structures of MOFs provide well-defined microenvironments for various guest species (molecules, clusters, and particles), and the ability to probe confined guest species is crucial for understanding the interaction and synergy between the host and guest components^88–90^. In most cases, guest species are not periodically distributed in the framework of the host material, which makes it difficult to precisely locate them using diffraction data; real-space imaging is therefore a better choice in this regard. However, the precise positioning of guest species requires the structure of the host material to be well preserved during imaging, which was not possible before the development of suitable low-dose electron microscopy techniques.

**Fig. 6:**
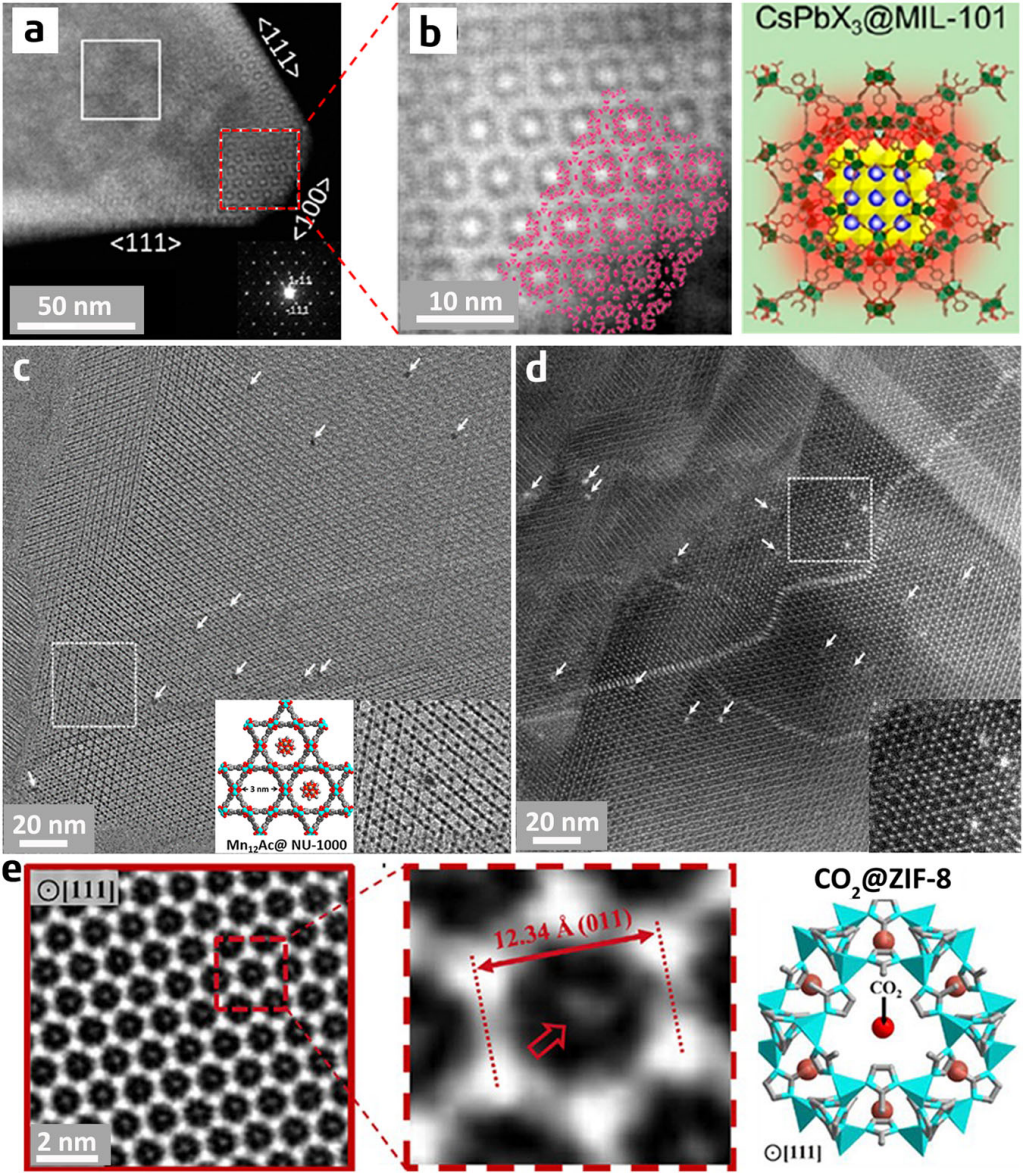
Guest species in MOFs. **a**, **b** HAADF-STEM image and schematic illustration of perovskite CsPbI_3_ quantum dots confined in MIL-101 (adapted with permission from ref. ^90^. © 2019 American Chemical Society). **c** HR-TEM and **d** HAADF-STEM images of single-molecular magnet, Mn_12_Ac, isolated by the porous matrix of NU-1000. White arrows indicated the encapsulated Mn_12_Ac clusters selectively residing in the hexagonal channels. The insets show the schematic illustration and the enlarged images of the highlighted square areas (adapted with permission from ref. ^34^. © 2019 American Chemical Society). **e** Cryo-TEM image and simulated structure of ZIF-8 with adsorbed CO_2_ molecules. The arrow indicates the adsorbed CO_2_ molecule (adapted with permission from ref. ^91^. © 2019 Elsevier).

Meledina et al. used atomic layer deposition to prepare Pt nanoparticles inside the pores of MIL-101 and observed the resulting composite material with ADF-STEM^46^. Their images revealed that crystalline or partially crystallized Pt particles were formed inside both large (3.4 nm) and small (2.9 nm) cages of MIL-101. Cha et al. used a simple method to produce, in situ, all-inorganic perovskite, CsPbX_3_ (X = I, Br, Cl) inside MIL-101 crystals at room temperature and conducted HAADF-STEM to characterize the resulting composite material (Fig. 6a, b)^90^. They attributed the unusual bright contrast observed in the HAADF-STEM image to CsPbI_3_ quantum dots residing in the mesoporous cages of MIL-101. They claimed that MIL-101 not only functioned as a nanoreactor for the formation of uniform perovskite quantum dots but also improved the stability of these dots.

Aulakh et al. encapsulated a single-molecule magnet, [Mn_12_O_12_(O_2_CCH_3_)_16_(H_2_O)_4_] or Mn_12_Ac for short, in the mesoporous channels of MOF NU-1000^34^. The resulting Mn_12_Ac@NU-1000 composite exhibited improved stabilities with the preservation of magnetic bistability. In order to obtain direct evidence for the isolation of Mn_12_Ac in the porous matrix of NU-1000, they characterized Mn_12_Ac@NU-1000 composite using HR-TEM in a collaboration with Liu and Han^34^. The obtained HR-TEM images indicated that the composite retained the highly ordered structure of NU-1000, comprising alternating hexagonal and trigonal channels and widely distributed discrete Mn_12_Ac clusters (as indicated by the white arrows in Fig. 6c). The enlarged images confirmed that each Mn_12_Ac cluster was selectively and precisely located in the hexagonal channel rather than the trigonal channel. This result is in good agreement with the experimental design based on the size of the Mn_12_Ac cluster (~1.6 nm) and the diameters of hexagonal (3.1 nm) and trigonal (1.2 nm) channels. This is the first study to utilize high-resolution electron microscopy to locate molecular guests in a MOF-based host material. Further, HAADF-STEM images obtained in a subsequent study confirmed the successful isolation of Mn_12_Ac clusters by the porous matrix of NU-1000 with improved image contrast (Fig. 6d; unpublished).

Using low-dose TEM performed at a cryogenic temperature (cryo-TEM), Li et al. reported that they observed, for the first time, physically adsorbed CO_2_ molecules in the pores of ZIF-8^91^. In their experiment, they froze the specimen at the liquid nitrogen temperature to prevent CO_2_ desorption and then used a total electron dose of 7 e^−^ Å^−2^ for HR-TEM imaging to avoid beam damage to the ZIF-8 structure. They acquired HR-TEM images along the [111] and [100] zone axes of ZIF-8. In both directions, they observed bright contrast in the centre of each pore and assigned this bright contrast to the CO_2_ molecules adsorbed at the centres of the 6-ring window and the 4-ring window, respectively. According to the density functional theory calculations, the two distinct CO_2_ binding sites identified from HR-TEM are both energetically favourable. The images and the proposed structural model of the [111] direction are shown in Fig. 6e.

Using cryo-TEM to image gas molecules adsorbed in MOFs is indeed a good strategy, because the cryogenic temperature effectively prevents gas desorption, while at the same time improving the electron dose tolerance of the MOF. However, it should be noted that in Li’s work, individual Zn atomic columns were not resolved in the image, although the image resolution was sufficient, suggesting that the image processing (CTF-correction) might not be perfectly accurate. Given that imperfect image processing can lead to the generation of artefacts in the processed image, more evidence is needed to confirm that the observed bright contrast in the pores is not an artefact.

## Outlook

In the previous sections, we demonstrated that the development of novel low-dose electron microscopy techniques has enabled atomic-resolution imaging of MOFs, with a number of bulk and local MOF structures unravelled by HR-(S)TEM. In this section, we share some of our experiences, perspectives, and outlook on this subject.

### Electron dose tolerance of MOFs

Accurately determining the electron dose tolerance of a MOF is difficult, as it varies with imaging conditions (e.g. accelerating voltage, imaging mode, and operation temperature) and the size and orientation of the MOF crystal. We investigated the stability of a few widely studied MOFs, including MOF-5, UiO-66, ZIF-8, NU-1000, and MIL-101, under a parallel electron beam at 300 kV and room temperature by acquiring a time series of selected-area electron diffraction patterns from sub-micron-sized MOF crystals to monitor the fading of diffraction spots. We define the cumulative electron dose that causes all high-frequency diffraction spots (*d* < 2 Å) to disappear as the threshold of structural damage. Under such circumstances, the electron dose tolerances of the studied MOFs are determined to be 5–30 e^−^ Å^−2^. Interestingly, for a given MOF, when the crystal is oriented along a main zone axis, especially along the direction of the primary channels, it can apparently withstand more electron doses compared with an off-axis orientation. Lowering the imaging temperature from the room temperature to the liquid nitrogen temperature can increase the electron dose tolerance of a MOF by several times. We also note that mesoporous MOFs with large unit cells, such as NU-1000 and MIL-101, undergo structural shrinkage at very low doses (<10 e^−^ Å^−2^), thereby quickly losing atomic-scale structural ordering; meanwhile, such MOFs can retain the ordered arrangement of the pores even at high doses up to more than 100 e^−^ Å^−2^. Therefore, for these MOFs, it is relatively easy to obtain images with nanometre resolution but difficult to achieve atomic-resolution imaging^40,46,48,92^.

The change of the diffraction pattern is commonly used as a criterion of beam damage. However, it is worth noting that this method is in principle only suitable for evaluating the beam tolerance of the bulk structure, whereas the local, non-periodic structures may be more beam sensitive. For example, previous studies indicate that the electron beam damage at crystal surfaces is more severe than at the bulk^14^. Unfortunately, there is no better way to determine the precise beam tolerance of individual local structural feature. Therefore, we recommend the use of lowest possible (lower than the threshold determined for the bulk structure) electron dose for imaging local structures; meanwhile, it is better to acquire multiple images from different specimens to confirm that the observed local structural feature is inherent and not randomly generated due to beam damage.

### Criteria for HR-TEM image processing

Since the HR-TEM image contrast varies with the defocus value, the raw image is usually processed by CTF-correction to make it more interpretable. Thus, a key question is raised: what are the criteria for determining whether the image is processed correctly? Unfortunately, we have to face some uncertainty in CTF-correction, because the absolute defocus value cannot be determined very precisely, but only approximately. Our common practices (and our recommendations) are as follows: we acquire images from the thinnest area of the specimen and select the image with an optimal focusing condition (defocus value < 300 nm) for image processing; we calculate a series of images through CTF-correction, using different defocus values (±100 nm around the approximately determined defocus with 1 nm interval)^35^; meanwhile, we simulate the electrostatic potential map of the studied material, based on the structure determined by X-ray diffraction or modelling; finally, we compare the ‘bulk structure’ presented in the CTF-corrected images with the simulated electrostatic potential map, and choose the image with the highest degree of matching as the ‘correctly processed’ image for further analyses of ‘local’ structures. In short, we recommend using the known ‘bulk’ structure as a standard to evaluate image processing: the processed image should be reasonably matched to the simulated electrostatic potential, correctly displaying structural features (e.g. the geometry of metal clusters, the separation distance between metal atoms, and the shape of the channels) within the resolution limit. For a completely unknown structure, there is no other option but to use the approximately determined defocus to perform CTF-correction.

### iDPC-STEM

Compared with commonly used dark-field STEM (ADF-STEM and HAADF-STEM), iDPC-STEM utilizes the incident electrons more efficiently, which combined with the integration progress produces better SNR under the low-dose conditions required for materials that are sensitive to electron beams. On the other hand, iDPC-STEM images are easier to interpret than HR-TEM images. We successfully imaged various guest molecules, including volatile organic compounds and MoO_3_ clusters, in the micropores of zeolites using iDPC-STEM, showing that iDPC-STEM images are almost directly interpretable without the need for image processing^53^.

**Fig. 7:**
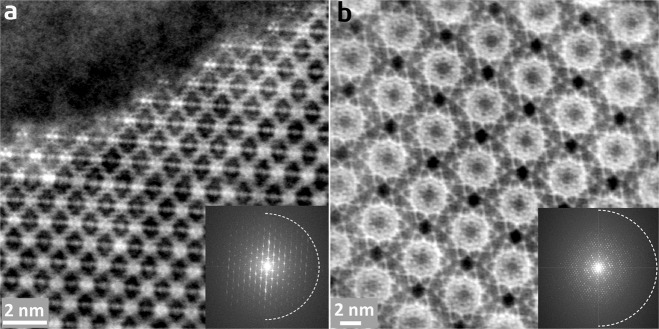
Atomic-resolution iDPC-STEM images of MOFs. **a** UiO-66 and **b** MIL-101. Insets are the corresponding FFT patterns, in which the dashed circle represent information transfer of 1.5 Å in **a** and 2.0 Å in **b**. The images are used by courtesy of Anna Carlsson and Daniel Stroppa, Thermo Fisher Scientific.

In 2019, we reported the first application of iDPC-STEM in MOF imaging^36^. In that study, we used both low-dose HR-TEM and iDPC-STEM to observe the surface structures of MIL-101; we found that iDPC-STEM exhibited better image contrast but poorer resolution than HR-TEM (4.2 vs. 2.5 Å). Subsequently, Deng et al. utilized iDPC-STEM to study beam-induced structural evolvement of MIL-101 and they reported a similar resolution of 4.7 Å^93^. Recently, we realized that by optimizing imaging conditions to minimize the beam damage, iDPC-STEM can generate images of MOFs with the same high resolution as low-dose HR-TEM: ~1.5 Å for UiO-66 (Fig. 7a) and ~2.0 Å for MIL-101 (Fig. 7b). An independent study recently reported similar results^94^. With such a high resolution, individual Zr columns and organic BDC linkers in UiO-66 can be clearly identified (Fig. 7a). More importantly, these directly acquired phase-contrast images showed a perfect match with the projected MOF structures without complicated image processing. The images presented in Fig. 7 were acquired at 200 kV with a total electron dose higher than 100 e^−^ Å^−2^, thereby suggesting that the damage mechanisms in STEM and TEM are different.

Overall, iDPC-STEM offers a good balance between the structural preservation of the specimen (resolution), the SNR of the image, and the contrast of organic species. Therefore, we predict that iDPC-STEM will be increasingly used in MOF imaging.

### Limitation of projection images

Because (S)TEM images are projection images, they lack resolving ability in the projection direction. This limitation requires that the structure features under study must be aligned or concentrated along the projection direction to produce sufficient contrast for recognition. For example, we can see the ‘missing-linker’ and ‘missing-cluster’ defects in UiO-66 with HR-TEM because the defective unit cells are assembled into ordered domains. If the defective unit cells are completely randomly distributed in the crystal, unless the (S)TEM specimen is made one-unit-cell thin, we cannot identify them in the image due to lack of contrast. Therefore, we cannot rule out the existence of randomly distributed local structures even if they are not evident in the (S)TEM images.

### (S)TEM specimen preparation

High-resolution (S)TEM imaging requires the specimen to be very thin, ideally <100 nm. This requirement makes (S)TEM specimen preparation crucial for imaging large crystals. Since MOFs are traditionally considered unsuitable for (S)TEM characterization due to their extreme beam sensitivity, no attention has been given to their specimen preparation. Almost all current (S)TEM studies on MOFs were conducted by directly imaging nano-sized MOF crystals without the need for specimen preparation. However, not all MOFs can be synthesized in the form of nanocrystals, while large MOF crystals are preferred in practical applications because they are easy to handle. Given that high-resolution imaging of MOFs has become feasible with the development of low-dose HR-TEM and iDPC-STEM, it is time to explore specimen preparation methods for MOFs to extend the application of new imaging techniques to large MOF crystals. Unfortunately, MOFs are sensitive not only to the electron beam but also to other forces, and they are therefore susceptible to structural damage during common TEM specimen preparation processes. For example, certain MOF crystals can be amorphized by grinding and crushing. Therefore, preparing thin specimens suitable for (S)TEM imaging from large MOF crystals is a challenge to be solved.

Ultramicrotomy is an effective technique for preparing (S)TEM specimens, but not suitable for MOFs that are structurally or chemically unstable upon exposure to water, because it needs to use the surface tension of water to support the thin slices (50–100 nm) that are cut from crystals embedded in epoxy resin, and then use a TEM grid to collect them. Ultramicrotomy has a high efficiency, producing a large number of specimen at a time. However, each specimen has a random crystal orientation, so a zone axis alignment process is required during imaging, which is similar to the case of powder samples.

Focused-ion beam (FIB) is another common method for preparing (S)TEM specimens. The unique advantage of FIB is that it allows site-specific and orientation-specific extraction of the specimen from a bulk crystal with nanometre-level precision. FIB is commonly used to cut ‘hard’ materials, and it is generally considered unsuitable for ‘soft’ or unstable materials, such as MOFs, because the ion beam can easily cause damages to their structures. Recently, we found that performing FIB at cryogenic temperatures (cryo-FIB) can effectively lessen the structural damage during ion beam milling. Using cryo-FIB, we successfully extracted a thin slice from a micron-sized MOF HKUST-1 crystal without structural damage (HR-TEM image with a resolution up to 1.2 Å can be obtained); in contrast, performing FIB at room temperature (conventional FIB) completely destroyed its structure^95^. This result indicates that the stability of MOFs under the ion beam may be greatly improved at cryogenic temperatures, and that cryo-FIB provides a promising solution to the challenge of preparing (S)TEM specimens for various MOFs and other sensitive materials.

## References

[1] Li H, Eddaoudi M, O’Keeffe M, Yaghi OM (1999). Design and synthesis of an exceptionally stable and highly porous metal-organic framework. Nature.

[2] Furukawa H, Cordova KE, O’Keeffe M, Yaghi OM (2013). The chemistry and applications of metal-organic frameworks. Science.

[3] Fang Z, Bueken B, De Vos DE, Fischer RA (2015). Defect-engineered metal–organic frameworks. Angew. Chem. Int. Ed..

[4] Song Q (2012). Zeolitic imidazolate framework (ZIF-8) based polymer nanocomposite membranes for gas separation. Energy Environ. Sci..

[5] Jin C, Lin F, Suenaga K, Iijima S (2009). Fabrication of a freestanding boron nitride single layer and its defect assignments. Phys. Rev. Lett..

[6] Willhammar T (2012). Structure and catalytic properties of the most complex intergrown zeolite ITQ-39 determined by electron crystallography. Nat. Chem..

[7] Wiktor C (2017). Transmission electron microscopy on metal-organic frameworks—a review. J. Mater. Chem. A.

[8] Chen Q (2020). Imaging beam-sensitive materials by electron microscopy. Adv. Mater..

[9] Zhu L (2013). Direct observations of the MOF (UiO-66) structure by transmission electron microscopy. CrystEngComm.

[10] Song K (2020). Atomic-resolution imaging of halide perovskites using electron microscopy. Adv. Energy Mater..

[11] Garcia A (2014). Analysis of electron beam damage of exfoliated MoS_2_ sheets and quantitative HAADF-STEM imaging. Ultramicroscopy.

[12] Han Y (2009). A tri-continuous mesoporous material with a silica pore wall following a hexagonal minimal surface. Nat. Chem..

[13] Ugurlu O (2011). Radiolysis to knock-on damage transition in zeolites under electron beam irradiation. Phys. Rev. B.

[14] Egerton RF, Li P, Malac M (2004). Radiation damage in the TEM and SEM. Micron.

[15] Egerton RF (2019). Radiation damage to organic and inorganic specimens in the TEM. Micron.

[16] Zuo JM (2003). Atomic resolution imaging of a carbon nanotube from diffraction intensities. Science.

[17] Hashimoto A (2004). Direct evidence for atomic defects in graphene layers. Nature.

[18] Egerton RF (2012). Mechanisms of radiation damage in beam-sensitive specimens, for TEM accelerating voltages between 10 and 300 kV. Microsc. Res. Tech..

[19] Ghosh S (2019). Electron-beam-damage in metal organic frameworks in the TEM. Microsc. Microanal..

[20] Wiktor C (2012). Imaging of intact MOF-5 nanocrystals by advanced TEM at liquid nitrogen temperature. Microporous Mesoporous Mater..

[21] Ogata AF (2020). Direct observation of amorphous precursor phases in the nucleation of protein–metal–organic frameworks. J. Am. Chem. Soc..

[22] Zhu Y (2017). Unravelling surface and interfacial structures of a metal–organic framework by transmission electron microscopy. Nat. Mater..

[23] Lebedev OI (2005). First direct imaging of giant pores of the metal−organic framework MIL-101. Chem. Mater..

[24] Milazzo A-C (2005). Active pixel sensor array as a detector for electron microscopy. Ultramicroscopy.

[25] Jin L, Bilhorn R (2010). Performance of the DDD as a direct electron detector for low dose electron microscopy. Microsc. Microanal..

[26] Li X (2013). Electron counting and beam-induced motion correction enable near-atomic-resolution single-particle cryo-EM. Nat. Methods.

[27] Xuong NH (2007). Future directions for camera systems in electron microscopy. Methods Cell Biol..

[28] Jin L (2008). Applications of direct detection device in transmission electron microscopy. J. Struct. Biol..

[29] Bartesaghi A (2015). 2.2 Å resolution cryo-EM structure of β-galactosidase in complex with a cell-permeant inhibitor. Science.

[30] Zhang D (2018). Atomic-resolution transmission electron microscopy of electron beam–sensitive crystalline materials. Science.

[31] Shen K (2018). Ordered macro-microporous metal-organic framework single crystals. Science.

[32] Yang F (2018). Morphological map of ZIF-8 crystals with five distinctive shapes: feature of filler in mixed-matrix membranes on C_3_H_6_/C_3_H_8_ separation. Chem. Mater..

[33] Zhang Q (2019). Oxygen-assisted cathodic deposition of zeolitic imidazolate frameworks with controlled thickness. Angew. Chem. Int. Ed..

[34] Aulakh D (2019). Direct imaging of isolated single-molecule magnets in metal–organic frameworks. J. Am. Chem. Soc..

[35] Liu L (2019). Imaging defects and their evolution in a metal–organic framework at sub-unit-cell resolution. Nat. Chem..

[36] Li X (2019). Direct imaging of tunable crystal surface structures of MOF MIL-101 using high-resolution electron microscopy. J. Am. Chem. Soc..

[37] Wang J (2020). Engineering effective structural defects of metal–organic frameworks to enhance their catalytic performances. J. Mater. Chem. A.

[38] Díaz-García M, Mayoral Á, Díaz I, Sánchez-Sánchez M (2014). Nanoscaled M-MOF-74 materials prepared at room temperature. Cryst. Growth Des..

[39] Li P (2018). Hierarchically engineered mesoporous metal-organic frameworks toward cell-free immobilized enzyme systems. Chem.

[40] Leus K (2016). Atomic layer deposition of Pt nanoparticles within the cages of MIL-101: a mild and recyclable hydrogenation catalyst. Nanomaterials.

[41] Pennycook SJ, Jesson DE (1991). High-resolution Z-contrast imaging of crystals. Ultramicroscopy.

[42] Batson PE, Dellby N, Krivanek OL (2002). Sub-ångstrom resolution using aberration corrected electron optics. Nature.

[43] Liu Z (2013). A review of fine structures of nanoporous materials as evidenced by microscopic methods. Microscopy.

[44] Han L (2014). Structures of silica-based nanoporous materials revealed by microscopy. Z. Anorg. Allg. Chem..

[45] Li CM, Zhang Q, Mayoral A (2020). Ten years of aberration corrected electron microscopy for ordered nanoporous materials. ChemCatChem.

[46] Meledina M (2016). Direct imaging of ALD deposited Pt nanoclusters inside the giant pores of MIL-101. Part. Part. Syst. Charact..

[47] Li P (2017). Bottom-up construction of a superstructure in a porous uranium-organic crystal. Science.

[48] Platero-Prats AE (2017). Bridging zirconia nodes within a metal–organic framework via catalytic Ni-hydroxo clusters to form heterobimetallic nanowires. J. Am. Chem. Soc..

[49] Ikuno T (2017). Methane oxidation to methanol catalyzed by Cu-oxo clusters stabilized in NU-1000 metal–organic framework. J. Am. Chem. Soc..

[50] Zheng J (2018). Exceptional fluorocarbon uptake with mesoporous metal–organic frameworks for adsorption-based cooling systems. ACS Appl. Energy Mater..

[51] Lazić I, Bosch EGT, Lazar S (2016). Phase contrast STEM for thin samples: integrated differential phase contrast. Ultramicroscopy.

[52] Wu J (2020). Metal-organic framework for transparent electronics. Adv. Sci..

[53] Liu L (2020). Direct imaging of atomically dispersed molybdenum that enables location of aluminum in the framework of zeolite ZSM-5. Angew. Chem. Int. Ed..

[54] Yücelen E, Lazić I, Bosch EGT (2018). Phase contrast scanning transmission electron microscopy imaging of light and heavy atoms at the limit of contrast and resolution. Sci. Rep..

[55] Férey G (2005). A chromium terephthalate-based solid with unusually large pore volumes and surface area. Science.

[56] Deng H (2012). Large-pore apertures in a series of metal-organic frameworks. Science.

[57] Hmadeh M (2012). New porous crystals of extended metal-catecholates. Chem. Mater..

[58] Feng D (2015). Stable metal-organic frameworks containing single-molecule traps for enzyme encapsulation. Nat. Commun..

[59] Mayoral A (2015). Atomic observations of microporous materials highly unstable under the electron beam: the cases of Ti-doped AlPO_4_-5 and Zn–MOF-74. ChemCatChem.

[60] Wu H (2019). MXene derived metal–organic frameworks. J. Am. Chem. Soc..

[61] Amirjalayer S, Tafipolsky M, Schmid R (2014). Surface termination of the metal-organic framework HKUST-1: a theoretical investigation. J. Phys. Chem. Lett..

[62] McGuire CV, Forgan RS (2015). The surface chemistry of metal–organic frameworks. Chem. Commun..

[63] Shoaee M, Agger JR, Anderson MW, Attfield MP (2008). Crystal form, defects and growth of the metal organic framework HKUST-1 revealed by atomic force microscopy. CrystEngComm.

[64] Summerfield A, Cebula I, Schröder M, Beton PH (2015). Nucleation and early stages of layer-by-layer growth of metal organic frameworks on surfaces. J. Phys. Chem. C.

[65] Kumar A, Banerjee K, Foster AS, Liljeroth P (2018). Two-dimensional band structure in honeycomb metal–organic frameworks. Nano Lett..

[66] Moh PY, Cubillas P, Anderson MW, Attfield MP (2011). Revelation of the molecular assembly of the nanoporous metal organic framework ZIF-8. J. Am. Chem. Soc..

[67] Slater B, Ling S (2017). Look but don’t touch. Nat. Mater..

[68] Ben, S., Sanliang, L., Martin, H. & Christoph G. S. On the (110) surface structure of ZIF-8. *figshare*10.6084/m6089.figshare.4788910 (2017).

[69] Yao M-S (2019). Gas transport regulation in a MO/MOF interface for enhanced selective gas detection. J. Mater. Chem. A.

[70] Koo W-T, Jang J-S, Kim I-D (2019). Metal-organic frameworks for chemiresistive sensors. Chem.

[71] Seoane B (2015). Metal–organic framework based mixed matrix membranes: a solution for highly efficient CO_2_ capture?. Chem. Soc. Rev..

[72] Ma X (2018). Highly compatible hydroxyl-hunctionalized microporous polyimide-ZIF-8 mixed matrix membranes for energy efficient propylene/propane separation. ACS Appl. Nano Mater..

[73] Yanai N, Sindoro M, Yan J, Granick S (2013). Electric field-induced assembly of monodisperse polyhedral metal–organic framework crystals. J. Am. Chem. Soc..

[74] Avci C (2019). Template-free, surfactant-mediated orientation of self-assembled supercrystals of metal–organic framework particles. Small.

[75] Férey G (2004). A hybrid solid with giant pores prepared by a combination of targeted chemistry, simulation, and powder diffraction. Angew. Chem. Int. Ed..

[76] Mayoral A, Mahugo R, Sánchez-Sánchez M, Díaz I (2017). Cs-corrected STEM imaging of both pure and silver-supported metal-organic framework MIL-100(Fe). ChemCatChem.

[77] Yanai N, Granick S (2012). Directional self-assembly of a colloidal metal–organic framework. Angew. Chem. Int. Ed..

[78] Dissegna S (2018). Defective metal-organic frameworks. Adv. Mater..

[79] Sholl DS, Lively RP (2015). Defects in metal–organic frameworks: challenge or opportunity?. J. Phys. Chem. Lett..

[80] Ma, Y. et al. *Microscopy of Nanoporous Crystals*. 1431–1432 (Springer International Publishing, 2019).

[81] Valenzano L (2011). Disclosing the complex structure of UiO-66 metal organic framework: a synergic combination of experiment and theory. Chem. Mater..

[82] Shearer GC (2014). Tuned to perfection: ironing out the defects in metal–organic framework UiO-66. Chem. Mater..

[83] Wu H (2013). Unusual and highly tunable missing-linker defects in zirconium metal–organic framework UiO-66 and their important effects on gas adsorption. J. Am. Chem. Soc..

[84] Trickett CA (2015). Definitive molecular level characterization of defects in UiO-66 crystals. Angew. Chem. Int. Ed..

[85] Øien S (2014). Detailed structure analysis of atomic positions and defects in zirconium metal–organic frameworks. Cryst. Growth Des..

[86] Cliffe MJ (2014). Correlated defect nanoregions in a metal–organic framework. Nat. Commun..

[87] Schrimpf W (2018). Chemical diversity in a metal–organic framework revealed by fluorescence lifetime imaging. Nat. Commun..

[88] Lu G (2012). Imparting functionality to a metal–organic framework material by controlled nanoparticle encapsulation. Nat. Chem..

[89] Rösler C, Fischer RA (2015). Metal–organic frameworks as hosts for nanoparticles. CrystEngComm.

[90] Cha J-H (2019). Formation and encapsulation of all-inorganic lead halide perovskites at room temperature in metal–organic frameworks. J. Phys. Chem. Lett..

[91] Li Y (2019). Cryo-EM structures of atomic surfaces and host-guest chemistry in metal-organic frameworks. Matter.

[92] Mehdi BL (2017). Low-dose and in-painting methods for (near) atomic resolution STEM imaging of metal organic frameworks (MOFs). Microsc. Microanal..

[93] Zhou Y (2020). Local structure evolvement in MOF single crystals unveiled by scanning transmission electron microscopy. Chem. Mater..

[94] Shen B, Chen X, Shen K, Xiong H, Wei F (2020). Imaging the node-linker coordination in the bulk and local structures of metal-organic frameworks. Nat. Commun..

[95] Zhang D (2019). Cryo focused ion beam applications in high resolution electron microscopy studies of beam sensitive crystals. Microsc. Microanal..

